# The effect of the inclusion of trunk-strengthening exercises to a multimodal exercise program on physical activity levels and psychological functioning in older adults: secondary data analysis of a randomized controlled trial

**DOI:** 10.1186/s12877-022-03435-3

**Published:** 2022-09-10

**Authors:** Behnaz Shahtahmassebi, Jacinta Hatton, Jeffrey J. Hebert, Mark Hecimovich, Helen Correia, Timothy J. Fairchild

**Affiliations:** 1 Department of Exercise Physiology and Sport Injuries and Corrective Movements, Faculty of Sport Sciences, Ferdowsi University of Mashhad, Mashhad, Iran; 2 Disciplines of Psychology and Exercise Science, Murdoch University, Perth, Australia; 3 Faculty of Kinesiology, University of New Brunswick, Fredericton, Canada; 4 Athletic Training, University of Northern Iowa, Cedar Falls, IA USA

**Keywords:** Ageing, Accelerometer, Core Stability, Detraining, Exercise Therapy, Fear-of-falling, Mental health, Older adults, Psychological Functioning, Walking

## Abstract

**Background:**

Engaging in multimodal exercise program helps mitigate age-related decrements by improving muscle size, muscle strength, balance, and physical function. The addition of trunk-strengthening within the exercise program has been shown to significantly improve physical functioning outcomes. Whether these improvements result in improved psychological outcomes associated with increased physical activity levels requires further investigation. We sought to explore whether the inclusion of trunk-strengthening exercises to a multimodal exercise program improves objectively measured physical activity levels and self-reported psychological functioning in older adults.

**Method:**

We conducted a secondary analysis within a single-blinded parallel-group randomized controlled trial. Sixty-four healthy older (≥ 60 years) adults were randomly allocated to a 12-week walking and balance exercise program with (*n* = 32) or without (*n* = 32) inclusion of trunk strengthening exercises. Each program involved 12 weeks of exercise training, followed by a 6-week walking-only program (identified as detraining). Primary outcome measures for this secondary analysis were physical activity (accelerometry), perceived fear-of-falling, and symptoms of anxiety and depression.

**Results:**

Following the 12-week exercise program, no significant between-group differences were observed for physical activity, sedentary behaviour, fear-of-falling, or symptoms of anxiety or depression. Significant within-group improvements (adjusted mean difference [95%CI]; percentage) were observed in moderate-intensity physical activity (6.29 [1.58, 11.00] min/day; + 26.3%) and total number of steps per min/day (0.81 [0.29 to 1.33] numbers or + 16.3%) in trunk-strengthening exercise group by week 12. With respect to within-group changes, participants in the walking-balance exercise group increased their moderate-to-vigorous physical activity (MVPA) (4.81 [0.06 to 9.56] min/day; + 23.5%) and reported reduction in symptoms of depression (-0.26 [-0.49 to -0.04] points or -49%) after 12 weeks of the exercise program. The exercise-induced increases in physical activity levels in the trunk-strengthening exercise group were abolished 6-weeks post-program completion. While improvements in physical activity levels were sustained in the walking-balance exercise group after detraining phase (walking only).

**Conclusions:**

The inclusion of trunk strengthening to a walking-balance exercise program did not lead to statistically significant between-group improvements in physical activity levels or psychological outcomes in this cohort following completion of the 12-week exercise program.

**Trial registration:**

Australian and New Zealand Clinical Trials Registry (ACTRN12613001176752), registered on 28/10/2013.

**Supplementary Information:**

The online version contains supplementary material available at 10.1186/s12877-022-03435-3.

## Introduction

Multimodal exercise programs incorporating balance and resistance training increase muscle strength, balance, and physical functioning [1–4], thus reducing rate and risk of falling in older adults [5–7]. The fear-of-falling is a common and serious problem among older adults [8] which is considered as a complex phenomenon, affected by physical, physiological, psychological and functional factors [9]. Research has shown that fear-of-falling has been linked to increased risk of future falls through restriction in activities of daily living, cautious gait pattern and reductions in the walking speed, increased level of anxiety and depression, poor quality of life, and all-cause mortality [10–14]. The effect of balance and resistance training programs on reducing the fear-of-falling is, however, less clear [15]. While a systematic review on exercise-based interventions in older community-residing adults reported small to moderate reductions in fear-of-falling immediately post-intervention [15], this finding was based on data from studies deemed to be at high-risk of bias [15]. Fear-of-falling is associated with reduced physical activity levels [16, 17] through avoidance of activities of daily living which can lead to increased risk of mental conditions (e.g., anxiety, and depressive symptoms) among older people [18]. Whether reductions in the fear-of-falling result in increased physical activity remains to be determined [15, 17]. There are persistent longitudinal and bidirectional associations between physical activity levels and mental health [19]. The higher physical activity levels are associated with improved mental health and vice versa [19]. Considering the important role of physical activity in promoting healthy ageing [20, 21], improving mental health [19, 22], and preventing falls [7], this presents a significant public health concern and is an area requiring further research.

Previous research has demonstrated that age-related changes in trunk muscle size, strength, mobility of the lumbar spine, and spinal inclination can be considered as risk factors related to falls in older adults [23–27]. More recent studies have provided empirical support for the crucial role of trunk (core) muscles strengthening [2], due to the importance of these muscles in performing activities of daily living, balance, physical functioning, and falls prevention [26–30].

More recently, the novel findings of our randomized controlled trial [3] have confirmed that including trunk strengthening exercises into a multi-modal exercise program significantly improved trunk muscle size, strength, and multiple components of balance and functional ability in healthy older adults. Although previous research has shown that the trunk strengthening exercises are generally recommended in older populations with great physiological, physical and functional benefits [3, 28], the efficacy of trunk strengthening exercises on physical activity levels, sedentary behaviours, and psychological functioning (i.e., perceived fear-of-falling, anxiety, and depressive symptoms) in older adults require further investigation.

Therefore, we performed a secondary analysis using data from our randomized controlled trial [3] to explore whether the exercise-induced improvements in physical function and balance [3] would be translated into increased levels of physical activity [7, 31] and reductions in perceived fear-of-falling, anxiety, and depressive symptoms [22, 32, 33]. The first objective of this study was to examine changes in physical activity and sedentary behaviour in response to the multi-modal exercise program [3]. The second objective was to determine whether improvements in physical activity levels were associated with reductions in perceived fear-of-falling, anxiety, and depressive symptoms [17, 34]. The third objective of this study was to determine the effect of a subsequent 6-week detraining phase (an unsupervised walking program) on physical activity, sedentary behaviour, perceived fear-of-falling, anxiety, and depressive symptoms in healthy older adults.

## Methods

### Study design and setting

The present study is a secondary analysis of a single-blinded (participants) parallel group randomized controlled trial. The study design and protocol were approved by the Murdoch University Human Research Ethics Committee (Protocol No: 2013/140). All procedures were carried out in accordance with the principles of the Declaration of Helsinki [3]. Prior to study participation, participants provided written informed consent for study participation.

The trial was prospectively registered (ACTRN12613001176752) and reported in accordance with the CONSORT statement (http://www.consort-statement.org). The CONSORT 2010 checklist of this trial has been previously been published [3]. The baseline data, [27] as well as the changes in physical function from this trial have previously been published [3]. Briefly, participants were randomized to one of two exercise groups (1:1), either a walking-balance exercise program or a trunk strengthening exercise program, using a computer-generated block randomization list with random block sizes of 2, 4, or 6. Group allocation was conducted after completion of baseline testing, using sequentially numbered, opaque envelopes to ensure allocation concealment.

### Study participants

We recruited healthy individuals aged 60 years and older, who were able to participate in an 18-week exercise program [3]. Individuals were excluded from study participation if they i) had undergone lumbar spine surgery, ii) had any medical condition(s) or were taking prescribed medication that may have precluded safe participation in an exercise program according to a standardized adult pre-exercise screening tool [35] or iii) were unable to communicate in English [3]. Participants were recruited from the local community via posted flyers, announcements through local news outlets, and presentations at local retirement communities. From a total of 105 older individuals who were initially contacted and screened for inclusion in the study, 64 met the inclusion criteria and were recruited. The recruitment/data was collected from 02/2014–11/2015.

### Exercise programs

All exercise training sessions were supervised by the main instructor (BSH) using the same trainers. Each program involved 12 weeks of exercise training, followed by a 6-week walking-only program. One exercise program included walking and balance exercises only (active control group; walking-balance exercise program), while the other exercise program supplemented the walking and balance exercise with trunk-muscle strengthening/motor control exercises (exercise group; trunk strengthening exercise program). Participants attended three supervised sessions per week, and exercise difficulty was progressed over the course of the program. The details of exercise protocols for each group are presented in supplemental material (Table S1) [3].

### Detraining

Following completion of the 12-week exercise program, participants in both groups were instructed to continue with a walking-only program (45 min of continuous walking at approximately 60% of their maximum heart rate), three times per week, over the subsequent 6 weeks [3].

### Measurements

Demographic and anthropometric data were recorded at baseline along with all outcome measures. The primary outcome measures for this secondary analysis were objective measurements of physical activity and sedentary behavior, and self-report psychological functioning (perceived fear-of-falling, anxiety and depressive symptoms). Physical activity and sedentary behaviour (sedentary time; overall physical activity level, CPM; the total number of steps; light physical activity; moderate physical activity; vigorous physical activity; moderate to vigorous intensity physical activity; moderate‐to‐vigorous physical activity, MVPA) measured using a hip-worn, triaxial GT3X Actigraph accelerometer [36]. Self-report psychological functioning outcomes included perceived fear-of-falling (Falls Efficacy Scale-International) [37], anxiety symptoms (Geriatric Anxiety Inventory) [38], and depressive symptoms (Geriatric Depression Scale Short Form) [39]. All outcome measures were re-administered at week 6, week 12, and week 18. The details of anthropometric and demographic characteristics have been described previously [3].

### Physical activity and sedentary behavior

Physical activity and sedentary behavior were objectively measured using a hip-worn, triaxial GT3X Actigraph accelerometer (Actigraph, Pensacola, Florida, USA). Accelerometers continuously record positional changes and movements over a given period of time [36] and are a reliable and valid device for measuring physical activity and sedentary behavior [36, 40–43]. Each participant was instructed to wear the accelerometer, which was attached to an adjustable elastic band, on the right hip at all times for seven consecutive days, except during water-related activities (e.g., swimming or showering). The Actilife software version 6.13.3 (Actigraph, Pensacola, Florida, USA) was used to initialize, download, and process accelerometry data. Data were recorded in 60-s epochs [44] and to be included in the analysis, participants were required to wear the accelerometer for ≥ 4 days and at least 10 h of valid wear time per day [44, 45]. Although we collected triaxial accelerometer data, only vertical axis data are presented and adopted in the analysis since it is most sensitive to ambulatory movements (e.g., major activities like normal walking, fast walking, incline descent or ascent walking) [40, 46]. Non-wear time was calculated based on the Choi's non-wear algorithm [45]. This algorithm provides more accurate estimation of time spent in sedentary and active behaviors, particularly in populations with a high sedentary and low active behavior [45]. Freedson Adult (1998) [46] cut points were applied for sedentary (0–99 counts per minute), light (100–1951 counts per minute), moderate (1952–5724 counts per minute), vigorous (5725—9498 counts per minute), and moderate-to-vigorous physical activity (≥ 1952 counts per minute). Outcomes of interest included sedentary time (min/day), total number of steps per min/day, overall physical activity level (average counts per minute, CPM) on vertical axis, light (min/day), moderate (min/day), vigorous (min/day), and moderate-to-vigorous physical activity (MVPA) (min/day), which was calculated by summing the minutes spent in moderate and vigorous physical activity.

### Fear-of-falling

Perceived fear-of-falling was assessed using the 16-item Falls Efficacy Scale-International questionnaire, which has excellent internal consistency and test–retest reliability (α = 0.96 and ICC = 0.96 respectively) [37]. The 16-item Falls Efficacy Scale-International quantified participants’ falls-related self-efficacy (level of concern), or their confidence in performing various activities of daily living without falling [47]. Participants rated their perceived fear-of-falling when performing 16 activities of daily living (ADLs), on a four-point Likert scale (1 = not at all concerned to 4 = very concerned) [37]. Scores were then added to yield a total score (maximum score = 64). Total scores categorised based on low concern (16–19), moderate concern (20–27) and high concern (28–64) of falling [48].

### Anxiety symptoms

The 20-item Geriatric Anxiety Inventory [38] assessed anxiety symptoms. It has high internal consistency in healthy older adults (α = 0.91) [38], and relatively high validity, with a sensitivity of 75% and a specificity of 84% [38]. Participants rated items corresponding to specific anxiety symptoms like unhelpful worrying, restlessness, irritability, and somatic complaints over the past week; by either ticking agree (score of 0) or disagree (score of 1) on a dichotomous scale [38]. Item scores are then summed. The minimum possible total score is 0, and the highest possible total score is 20. Higher scores represent greater generalised anxiety levels in older adults, and total scores of 9 and above represent clinically significant symptoms of self-reported anxiety [38].

### Depression symptoms

The 15-item Geriatric Depression Scale [39] measured depressive symptoms in older adults. The 15-item Geriatric Depression Scale has moderate internal consistency (α = 0.79) [49] and relatively high validity, with a sensitivity of 81% and a specificity of 75% [50]. Each question corresponded to symptoms of depression like hopelessness, helplessness, fluctuations in energy levels, and changes in engagement in activities over a given week, and participants either indicated ‘yes’ or ‘no’ to each question on a dichotomous scale. All scores are then summed up. Total scores are categorised as ‘normal’ (0–4), ‘mild’ (5–8), ‘moderate’ (9–11) or ‘severe’ (12–15). Scores of 6 and above are indicative of depressive symptoms and warrant further medical investigation [51].

### Data analysis

Data management and statistical analyses were performed using IBM SPSS version 22.0 software (IBM Corp, Armonk, NY). The primary outcomes for this secondary analysis were physical activity and sedentary behaviours (wear time; sedentary time; total number of steps; overall physical activity level (average counts per minute, CPM) on vertical axis; light physical activity; moderate physical activity; vigorous physical activity; MPVA), perceived fear-of-falling (Falls Efficacy Scale-International), anxiety symptoms (Geriatric Anxiety Inventory) and depressive symptoms (Geriatric Depression Scale Short Form). The proportion of wear time (as percentage of total daily wear time) spent in sedentary behaviour, light physical activity; moderate physical activity; vigorous physical activity, and MPVA was calculated by dividing the sum of time for a given outcome (minutes per day) by total valid wear time (minutes per day). Treatment effects were estimated with separate, random-intercept linear mixed models for each outcome variable. Time [baseline (week 0), 6 weeks, 12 weeks, 18 weeks] and exercise group (trunk strengthening, walking-balance) were modelled as fixed effects. All differences were only adjusted for the baseline value of the outcome variables and there were no adjustments for other variables apart from baseline values. The hypothesis of interest was the group by time interaction, which was examined with pairwise comparisons of the estimated marginal means. Consistent with the intention-to-treat principle, the linear mixed models estimated values for missing data based on the available scores; therefore, all participants were included in the analyses. The level of significance was set at *p* ≤ 0.05.

## Results

**Fig. 1:**
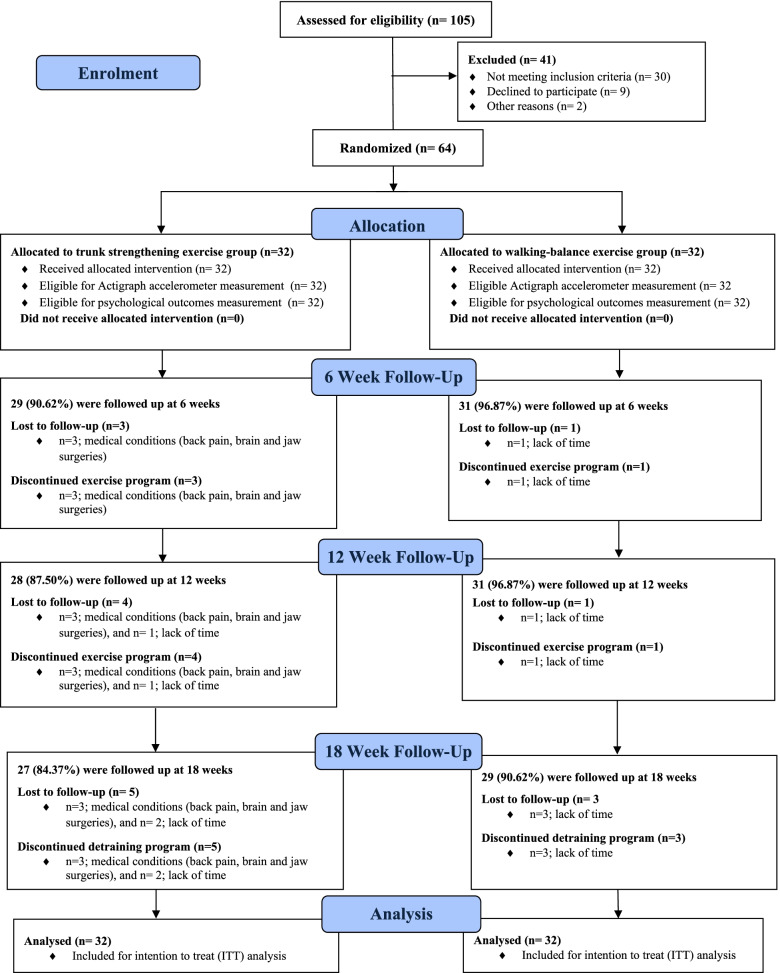
The study flow diagram for the Actigraph accelerometer and psychological functioning outcomes measurements

The baseline characteristics of participants are presented in Table 1 [3]. The study flow diagram for the Actigraph accelerometer and psychological functioning outcomes measurements is presented in Fig. 1.

**Table1:** Baseline characteristics of study’s participants stratified by exercise group

Characteristics	All (*n* = 64)	Trunk strengthening (*n* = 32)	Walking-balance (*n* = 32)
Age, years	69.8 ± 7.5	70.1 (7.7)	69.4 (7.3)
Sex n (%) female	38 (59.4)	18 (56.3)	20 (62.5)
Height, cm	165.1 (9.0)	166.5 (9.2)	163.8 (8.9)
Weight, kg	74.9 (14.8)	74.3 (14.0)	75.4 (15.8)
BMI, kg/m^2^	27.3 ± 4.7	26.6 (3.2)	28.1 (5.8)
Sitting height, cm	80.5 ± 5.0	81.5 (4.9)	79.5 (4.9)
Living status			
Lived with one or more than one person (%)	18 (28.1)	9 (28.1)	9 (28.1)
Lived alone (%)	46 (71.9)	23 (71.9)	23 (71.9)
Could drive (%)	62 (96.9)	32 (100)	30 (94)
Used glasses or contact lens (%)	55 (85.9)	25 (78.1)	30 (93.8)
Used hearing aids (%)	8 (12.5)	4 (12.5)	4 (12.5)
Used walking aid (%)	0	0	0
History of falls over past one month			
Falls n (%)	6 (9.4)	2 (6.3)	4 (12.5)
History of falls over past 12 months			
Falls (%)	12 (18.8)	6 (18.8)	6 (18.8)
Medications			
1–2 medications n (%)	27 (42.2)	14 (43.7)	13 (40.6)
3 medications or more n (%)	22 (12.5)	10 (31.3)	12 (37.6)
No medications n (%)	15 (23.4)	8 (25.0)	7 (21.8)
Self-reported physical activity			
Moderately active (1—2 times/week) n (%)	34 (53.1)	14 (43.7)	20 (62.5)
Very active (3 times/week) n (%)	28 (43.8)	16 (50.0)	12 (37.5)
Not very active (rarely leaves house) n (%)	2 (3.1)	2 (6.3)	0 (0)

Values are presented as mean (SD) or as number and percentage

*Note*. Adapted from “Trunk exercise training improves muscle size, strength, and function in older adults: A randomized controlled trial”, by Shahtahmassebi, B., Hebert, J. J., Hecimovich, M., & Fairchild, T. J (2019), *Scandinavian journal of medicine & science in sports*, 29(7), 980–991. https://doi.org/10.1111/sms.13415

### Changes in physical activity and sedentary behaviour in response to exercise program and detraining

The number (%) of participants and accumulated accelerometer valid wear time (days and hours) are presented in supplemental material (Table S2). The proportion of wear time spent in sedentary behaviour, light physical activity; moderate physical activity; vigorous physical activity, and MPVA is presented in supplemental material (Table S3). There were no significant differences in wear time between two exercise groups at all assessment time points (Supplemental material Table S4.

**Fig. 2:**
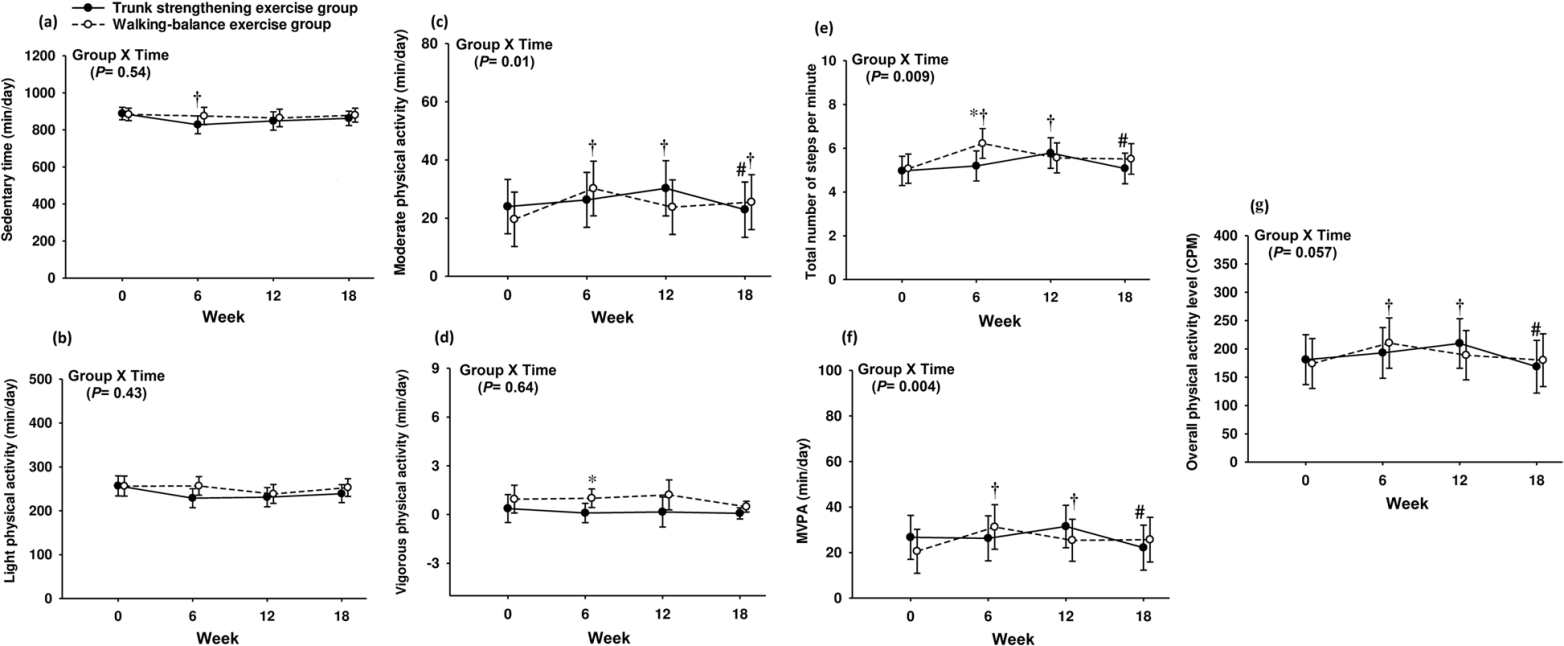
Changes in (**a**) sedentary behaviour, (**b**) light physical activity, (**c**) moderate activity, (**d**) vigorous activity, (**e**) moderate-vigorous activity, (**f**) total number of steps, (**g**) overall physical activity level in response to exercise program and detraining. All differences were estimated using linear mixed-effect models with random intercept and were adjusted for the baseline value of the outcome variables. Values are presented as mean values (95% CIs). * significant difference between groups at 0 ≤ 0.05, † significant difference from week 0 (baseline) at 0 ≤ 0.05, # significant difference from week 12 at 0 ≤ 0.05

At week 6, a significant time by group interaction was identified for physical activity outcomes (i.e., total number of steps per minute and vigorous physical activity) (Fig. 2; Supplemental material Table S4). Participants in the walking-balance exercise group showed significant between-group improvements (mean difference [95% CI] or percentage %) in total number of steps per min/day (1.02 [1.99 to 0.06] numbers or + 16.4%) and vigorous physical activity (0.90 [1.73 to 0.08] min/day or + 90%), compared to the trunk strengthening exercise group following week 6 (Fig. 2; Supplemental material Table S4). With respect to within-group changes, the trunk-strengthening exercise group showed a significant reduction in sedentary time (-60.3 [-111.2 to -9.4] min/day or -7%) between week 6 and baseline (Fig. 2; Supplemental material Table S4). There were significant improvements (mean difference [95% CI] or percentage %) in overall physical activity level (36.1 [14.8 to 57.4] CPM or + 20.7%), total number of steps per min/day (1.15 [0.65 to 1.64] numbers or + 23%), moderate physical activity levels (10.6 [5.74 to 15.4] min/day or + 54.3%), and MVPA (10.6 [4.85 to 16.4] min/day or + 52%) between week 6 and baseline in the walking-balance exercise program (Fig. 2; Supplemental material Table S4).

Following completion of the 12‐week exercise program, no significant time by group interaction were observed for all accelerometer measured outcomes (all *p* > 0.05). However, there were significant within-group improvements (mean difference [95% CI] or percentage %) in overall physical activity level (CPM) (28.8 [8.95 to 48.6] CPM or + 16%), total number of steps per min/day (0.81 [0.29 to 1.33] numbers or + 16.3%) and moderate physical activity levels (6.29 [1.58 to 11.00] min/day or + 26.3%) between week 12 and baseline in the trunk-strengthening program (Fig. 2; Supplemental material Table S4). Participants in the walking-balance exercise program demonstrated significant within-group improvement in MVPA (4.81 [0.06 to 9.56] min/day or + 23.5%) in week 12 compared to baseline (Fig. 2; Supplemental material Table S4).

Following the subsequent 6 weeks of detraining, there were no significant between group differences for physical activity and sedentary behaviour outcomes (all *p* > 0.05). Within-group changes showed participants in only the trunk strengthening exercise group experienced significant reductions (mean difference [95% CI] or percentage %) in overall physical activity level (average counts per minute) (-41.1 [-66.4 to -15.7] CPM or -19.6%), total number of steps per min/day (-0.70 [-1.25 to -0.15] numbers or -12.1%), moderate physical activity (-7.21 [-12.8 to -1.59] min/day or -24%), and MVPA (-9.32 [-14.5 to -4.06] min/day or -29%) at week 18 (Fig. 2; Supplemental material Table S4).

### Changes in psychological outcomes in response to exercise program and detraining

**Fig. 3:**
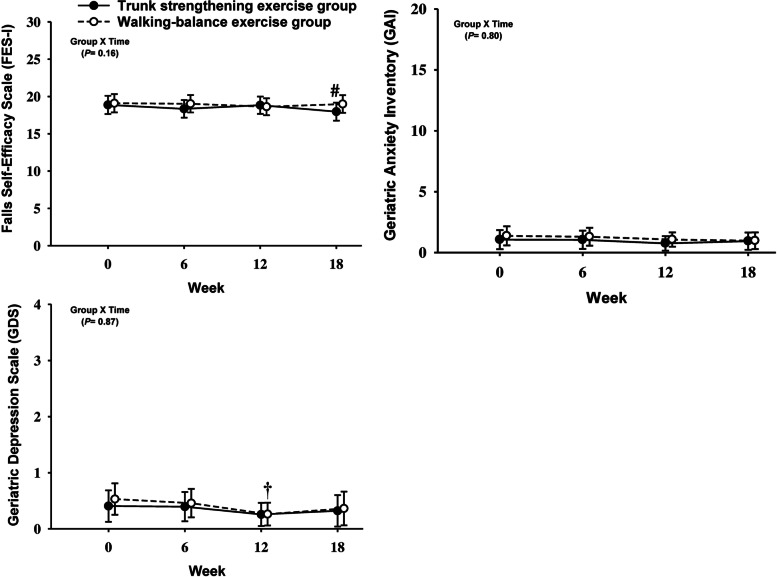
Changes in psychological outcomes in response to exercise program and detraining. All differences were estimated using linear mixed-effect models with random intercept and were adjusted for the baseline value of the outcome variables. Values are presented as mean values (95% CIs). * significant difference between groups at 0 ≤ 0.05, † significant difference from week 0 (baseline) at 0 ≤ 0.05, # significant difference from week 12 at 0 ≤ 0.05

There were no significant time by group interactions for perceived fear-of-falling, symptoms of anxiety and depression (Fig. 3; Supplemental material Table S5) following the 12-week exercise program. With respect to within-group changes, participants in the walking-balance exercise group experienced a significant reduction (mean difference [95% CI] or percentage %) in depression (-0.26 [-0.49 to -0.04] points or -49%) at week 12 (Fig. 3; Supplemental material Table S5).

Following the subsequent 6 weeks of detraining, there were no significant changes in perceived fear-of-falling, depression and anxiety between two exercise groups (all *p* > 0.05) (Fig. 3; Electronic Supplementary Material Table S5). Within-group changes showed participants in only the trunk strengthening exercise group experienced a significant reduction (mean difference [95% CI] or percentage %) in perceived fear-of-falling (0.86 [-1.70 to -0.03] points or -5%) in week 18 compared to week 12 (Fig. 3; Supplemental material Table S5).

## Discussion

Multimodal exercise programs have been shown to increase muscle size, strength, balance and physical function in older adults [1–3, 27] and reduce the rate and risk of falling [5, 6]. This study, a secondary analysis of a randomized controlled trial, aimed to assess whether the inclusion of trunk-strengthening exercises to a multimodal exercise program improved objectively measured physical activity levels and self-reported psychological functioning in older adults. The first objective of this study was to examine changes in physical activity and sedentary behaviour in response to the multi-modal exercise program incorporating trunk strengthening/motor control exercise. The second objective was to determine whether improvements in physical activity levels were associated with reductions in perceived fear-of-falling, anxiety, and depressive symptoms. The third objective was to determine the effect of a subsequent 6-week detraining phase (walking-only program) on perceived fear-of-falling, anxiety, and depression symptoms in healthy older adults. This study found that despite functional improvements and increased size and strength of the trunk musculature with the inclusion of trunk-exercises [3], this did not result in significant between-group differences in habitual physical activity levels, sedentary behaviour, or the fear-of-falling, symptoms of anxiety and depression in both exercise groups. There were no significant between-group differences either in the fear-of-falling, or symptoms of anxiety or depression following the 6-week detraining phase.

Participants in the walking-balance exercise group experienced significant increases in total number of steps per minute and vigorous physical activity at week 6, compared to the trunk strengthening exercise group. This is not surprising, considering that participants in the walking-balance exercise group walked 3 times (135 min per week) more than participants in the trunk strengthening exercise group (45 min per week). Notably, the trunk strengthening exercise group experienced a significant within-group reduction in sedentary time by 7% after week 6 of exercise program, and the walking-balance exercise group experienced significant within-group improvements in overall physical activity level (20.7%), total number of steps per minute (23%), moderate physical activity levels (54.3%), and MVPA (52%). Although there were no significant between group differences observed for all physical activity and sedentary behavior outcomes at week 12, there were significant within-group increases in overall physical activity level (CPM) (16%), total number of steps (16.3%), and moderate physical activity (30%) in the trunk strengthening exercise group and significant within-group increases in MVPA (23.5%) in walking-balance exercise group. Following the subsequent 6 weeks of detraining, only trunk strengthening exercise group experienced significant reductions in overall physical activity level (CPM) (19.6%), the total number of steps per minute/per day (12.1%), moderate physical activity (24%), and MVPA (29%). These changes also did not remain significantly above the baseline values. We speculate that when trunk strengthening/motor control exercises were excluded from the exercise program during detraining phase (only walking), participants in trunk strengthening exercise group experienced significant decline in physical activity levels following a 6-week detraining.

There were no significant between-group differences for perceived fear-of-falling, anxiety or depressive symptoms after the 12 weeks of exercise training. We found a significant within-group reduction in depression symptoms only in the walking-balance exercise group after 12 weeks. Overall, however, changes in psychological functioning were relatively modest, as we recruited only healthy older individuals who had no symptoms in the clinical range at baseline. Specifically, a Falls Self-Efficacy Scale (FES-I) score of 16–19 [37], a Geriatric Anxiety Inventory [17] score of 0 to 8 [38], a Geriatric Depression Scale (GDS) score of 0 to 4 [39], are considered within the normal range. It is likely that recruitment of older adults with higher perceived fear-of-falling [15], moderate/severe symptoms of anxiety [52] and/or depression [53] will lead to more prominent changes in outcomes. Following the subsequent 6 weeks of detraining (walking only), only participants in the trunk strengthening exercise group experienced a significant reduction in perceived fear-of-falling by 5% at week 18 (detraining) compared to week 12. Our results imply that despite being exposed to a reduced volume of training in the last 6 weeks (detraining), participants in the trunk strengthening group did not perceive their risk of falling as being different. While physical function did show slight decrements during the detraining phase [3], it remains to be determined whether this perception equated to the actual risk of falling.

The present study had several strengths. First, the study design was a randomized controlled design. Second, adherence rates for both trunk strengthening and walking-balance exercise groups were considerably high and dropout rates were low in both exercise groups. Third, we implemented valid and reliable outcome measures [3]*.* However, we acknowledge a number of limitations. Our study’s participants were mainly healthy and active older adults. Consequently, the current study’s findings may not generalise to other populations such as clinical populations [3]. We calculated the sample size (based on changes in trunk muscle morphology) of the main trial which has been previously reported [3], however this can be a limitation of our current study. The single-blinded design resulted in the assessments (physical activity, sedentary behaviour, and psychological functioning) being undertaken by the same individuals (BSH and JH) involved in the exercise training program which might be a potential effect of the acquisition on the assessments.

Future research should focus on broader, long-term outcomes including falls risk, incidence of falls and related injuries, other fall-related psychological concerns (e.g., low self-confidence, low self-esteem, reduced self-efficacy, and social isolation) following similar exercise programs. In addition, the benefits of this type of exercise program in clinical populations (i.e., sedentary, frail older adults, and musculoskeletal disorders) require further investigation. Furthermore, future research should focus on the double-blind method to minimize the effect of the acquisition on the assessments.

## Conclusion

In summary, although there were no significant between group differences, we found that participants in the trunk strengthening exercise group experienced i) minor significant within-group improvements in physical activity and minor reduction in sedentary behaviour, but ii) no significant reduction in perceived fear-of-falling, depression or anxiety compared to the baseline. Six weeks of detraining (walking program only) significantly decreased some physical activity outcomes (i.e., overall physical activity (CPM), total number of steps, moderate, MVPA) and caused significant reduction in perceived fear-of-falling in the trunk strengthening exercise group only.

## Supplementary Information

**Additional file 1: Table S1.** Detailed exercise protocol for each group.

**Additional file 2: Table S2.** Accumulated accelerometer valid wear time.

**Additional file 3: Table S3.** The proportion of wear time spent in sedentary behavior and different intensity levels of physical activity.

**Additional file 4: Table S4.** Changes in physical activity and sedentary behavior in response to the exercise program and detraining.

**Additional file 5: Table S5.** Changes in psychological outcomes in response to the exercise program and detraining.

## Data Availability

The dataset used and/or analyzed during the current study are not publicly available due to the confidentiality and anonymity of participants. However, the data should be available from the corresponding author after approval by the Murdoch University Human Research Ethics Committee.

## Abbreviations

CPM: Counts per minute
MVPA: Moderate-to-vigorous physical activity

## References

[1] Chodzko-Zajko WJ (2014). Exercise and physical activity for older adults. Kinesiol Rev (Champaign).

[2] Granacher U, Gollhofer A, Hortobagyi T, Kressig RW, Muehlbauer T (2013). The importance of trunk muscle strength for balance, functional performance, and fall prevention in seniors: a systematic review. Sports Med.

[3] Shahtahmassebi B, Hebert JJ, Hecimovich M, Fairchild TJ (2019). Trunk exercise training improves muscle size, strength, and function in older adults: a randomized controlled trial. Scand J Med Sci Sports.

[4] Briggs BC, Oursler KK. Pilot study of functional circuit exercise in older adults. Res Sports Med. 2021:1–6. 10.1080/15438627.2021.1966006 34402693

[5] Windle G, Hughes D, Linck P, Russell I, Woods B (2010). Is exercise effective in promoting mental well-being in older age? A systematic review. Aging Ment Health.

[6] Sherrington C, Fairhall NJ, Wallbank GK, Tiedemann A, Michaleff ZA, Howard K, Clemson L, Hopewell S, Lamb SE (2019). Exercise for preventing falls in older people living in the community. Cochrane Database Syst Rev.

[7] Sherrington C, Fairhall N, Kwok W, Wallbank G, Tiedemann A, Michaleff ZA, Ng CA, Bauman A (2020). Evidence on physical activity and falls prevention for people aged 65+ years: systematic review to inform the WHO guidelines on physical activity and sedentary behaviour. Int J Behav Nutr Phys Act.

[8] Scheffer AC, Schuurmans MJ, Van Dijk N, Van Der Hooft T, De Rooij SE (2008). Fear of falling: measurement strategy, prevalence, risk factors and consequences among older persons. Age Ageing.

[9] Hoang OTT, Jullamate P, Piphatvanitcha N, Rosenberg E (2017). Factors related to fear of falling among community-dwelling older adults. J Clin Nurs.

[10] de Souza LF, Canever JB, de Souza MB, Danielewicz AL, de Avelar NCP (2022). Association between fear of falling and frailty in community-dwelling older adults: a systematic review. Clin Interv Aging.

[11] Delbaere K, Sturnieks DL, Crombez G, Lord SR (2009). Concern about falls elicits changes in gait parameters in conditions of postural threat in older people. J Gerontol A Biol Sci Med Sci.

[12] Ellmers TJ, Cocks AJ, Young WR (2019). Evidence of a link between fall-related anxiety and high-risk patterns of visual search in older adults during adaptive locomotion. J Gerontol A Biol Sci Med Sci.

[13] Kim J-H, Bae SM (2020). Association between Fear of Falling (FOF) and all-cause mortality. Arch Intern Med.

[14] Gambaro E, Gramaglia C, Azzolina D, Campani D, Dal Molin A, Zeppegno P (2022). The complex associations between late life depression, fear of falling and risk of falls. a systematic review and meta-analysis. Ageing Res Rev..

[15] Kumar A, Delbaere K, Zijlstra GA, Carpenter H, Iliffe S, Masud T, Skelton D, Morris R, Kendrick D (2016). Exercise for reducing fear of falling in older people living in the community: cochrane systematic review and meta-analysis. Age Ageing.

[16] Howland J, Lachman ME, Peterson EW, Cote J, Kasten L, Jette A (1998). Covariates of fear of falling and associated activity curtailment. Gerontologist.

[17] Jefferis BJ, Iliffe S, Kendrick D, Kerse N, Trost S, Lennon LT, Ash S, Sartini C, Morris RW, Wannamethee SG (2014). How are falls and fear of falling associated with objectively measured physical activity in a cohort of community-dwelling older men?. BMC Geriatr.

[18] van Haastregt JC, Zijlstra GR, van Rossum E, van Eijk JTM, Kempen GI (2008). Feelings of anxiety and symptoms of depression in community-living older persons who avoid activity for fear of falling. Am J Geriatr Psychiatry.

[19] Steinmo S, Hagger-Johnson G, Shahab L (2014). Bidirectional association between mental health and physical activity in older adults: whitehall II prospective cohort study. Prev Med.

[20] Daskalopoulou C, Stubbs B, Kralj C, Koukounari A, Prince M, Prina AM (2017). Physical activity and healthy ageing: a systematic review and meta-analysis of longitudinal cohort studies. Ageing Res Rev.

[21] Bembom O, van der Laan M, Haight T, Tager I (2009). Leisure-time physical activity and all-cause mortality in an elderly cohort. Epidemiology.

[22] Strawbridge WJ, Deleger S, Roberts RE, Kaplan GA (2002). Physical activity reduces the risk of subsequent depression for older adults. Am J Epidemiol.

[23] Kasukawa Y, Miyakoshi N, Hongo M, Ishikawa Y, Noguchi H, Kamo K, Sasaki H, Murata K, Shimada Y (2010). Relationships between falls, spinal curvature, spinal mobility and back extensor strength in elderly people. J Bone Miner Metab.

[24] Kato K, Hatanaka Y (2020). The influence of trunk muscle strength on walking velocity in elderly people with sarcopenia. J Phys Ther Sci.

[25] Ito T, Sakai Y, Sugiura H, Kawai K, Morita Y, Yamazaki K (2021). Association between trunk muscle strength and fall risk in older men and women with lumbar spondylosis. Healthcare..

[26] Granacher U, Lacroix A, Roettger K, Gollhofer A, Muehlbauer T (2014). Relationships between trunk muscle strength, spinal mobility, and balance performance in older adults. J Aging Phys Act.

[27] Shahtahmassebi B, Hebert JJ, Hecimovich MD, Fairchild TJ (2017). Associations between trunk muscle morphology, strength and function in older adults. Sci Rep.

[28] Granacher U, Lacroix A, Muehlbauer T, Roettger K, Gollhofer A (2013). Effects of core instability strength training on trunk muscle strength, spinal mobility, dynamic balance and functional mobility in older adults. Gerontology.

[29] Golubić A, Šarabon N, Marković G (2021). Association between trunk muscle strength and static balance in older women. J Women Aging.

[30] Ito T, Sakai Y, Yamazaki K, Oikawa M, Morita Y (2019). Relationship between L4/5 lumbar multifidus cross-sectional area ratio and fall risk in older adults with lumbar spinal stenosis: a retrospective study. Geriatrics.

[31] Scott D, Johansson J, Gandham A, Ebeling PR, Nordstrom P, Nordstrom A (2021). Associations of accelerometer-determined physical activity and sedentary behavior with sarcopenia and incident falls over 12 months in community-dwelling Swedish older adults. J Sport Health Sci.

[32] Kendrick D, Kumar A, Carpenter H, Zijlstra GR, Skelton DA, Cook JR, Stevens Z, Belcher CM, Haworth D, Gawler SJ (2014). Exercise for reducing fear of falling in older people living in the community. Cochrane Database Syst Rev..

[33] Awick EA, Ehlers DK, Aguiñaga S, Daugherty AM, Kramer AF, McAuley E (2017). Effects of a randomized exercise trial on physical activity, psychological distress and quality of life in older adults. Gen Hosp Psychiatry.

[34] Legters K (2002). Fear of falling. Phys Ther.

[35] Norton K, Norton L. Pre exercise screening - Guide to the Australian adult pre-exercise screening system. illustrate. Exercise and Sports Science Australia, Fitness Australia and Sports Medicine Australia. Adelaide: Exercise and Sport Sceince Australia; 2011. p. 1–53.

[36] Santos-Lozano A, Marín PJ, Torres-Luque G, Ruiz JR, Lucía A, Garatachea N (2012). Technical variability of the GT3X accelerometer. Med Eng Phys.

[37] Yardley L, Beyer N, Hauer K, Kempen G, Piot-Ziegler C, Todd C (2005). Development and initial validation of the Falls Efficacy Scale-International (FES-I). Age Ageing.

[38] Pachana NA, Byrne GJ, Siddle H, Koloski N, Harley E, Arnold E (2007). Development and validation of the Geriatric Anxiety Inventory. Int Psychogeriatr.

[39] Sheikh JI, Yesavage JA. Geriatric Depression Scale (GDS). Recent evidence and development of a shorter version. Clin Gerontol. 1986;5(1-2):165–73.

[40] Sasaki JE, John D, Freedson PS (2011). Validation and comparison of ActiGraph activity monitors. J Sci Med Sport.

[41] Duncan MJ, Rowlands A, Lawson C, Leddington Wright S, Hill M, Morris M, Eyre E, Tallis J (2020). Using accelerometry to classify physical activity intensity in older adults: what is the optimal wear-site?. Eur J Sport Sci.

[42] Mañas A, del Pozo-Cruz B, García-García FJ, Guadalupe-Grau A, Ara I (2017). Role of objectively measured sedentary behaviour in physical performance, frailty and mortality among older adults: a short systematic review. Eur J Sport Sci.

[43] Copeland JL, Esliger DW (2009). Accelerometer assessment of physical activity in active, healthy older adults. J Aging Phys Act.

[44] Gorman E, Hanson HM, Yang PH, Khan KM, Liu-Ambrose T, Ashe MC (2014). Accelerometry analysis of physical activity and sedentary behavior in older adults: a systematic review and data analysis. Eur Rev Aging Phys Act.

[45] Choi L, Liu Z, Matthews CE, Buchowski MS (2011). Validation of accelerometer wear and nonwear time classification algorithm. Med Sci Sports Exerc.

[46] Freedson PS, Melanson E, Sirard J (1998). Calibration of the Computer Science and Applications. Inc accelerometer Med Sci Sports Exerc.

[47] Tinetti ME, Mendes de Leon CF, Doucette JT, Baker DI (1994). Fear of falling and fall-related efficacy in relationship to functioning among community-living elders. J Gerontol.

[48] Delbaere K, Close JC, Mikolaizak AS, Sachdev PS, Brodaty H, Lord SR (2010). The Falls Efficacy Scale International (FES-I) a comprehensive longitudinal validation study. Age Ageing.

[49] Friedman B, Heisel MJ, Delavan RL (2005). Psychometric properties of the 15-item geriatric depression scale in functionally impaired, cognitively intact, community-dwelling elderly primary care patients. J Am Geriatr Soc.

[50] Wancata J, Alexandrowicz R, Marquart B, Weiss M, Friedrich F (2006). The criterion validity of the geriatric depression scale: a systematic review. Acta Psychiatr Scand.

[51] Greenberg SA (2007). How to try this: the geriatric depression scaleshort form. Am J Nurs.

[52] Herring MP, O’connor PJ, Dishman RK (2010). The effect of exercise training on anxiety symptoms among patients: a systematic review. Arch Intern Med.

[53] Blumenthal JA, Babyak MA, Moore KA, Craighead WE, Herman S, Khatri P, Waugh R, Napolitano MA, Forman LM, Appelbaum M (1999). Effects of exercise training on older patients with major depression. Arch Intern Med.

