# Supplementary material for: The effect of the inclusion of trunk-strengthening exercises to a multimodal exercise program on physical activity levels and psychological functioning in older adults: secondary data analysis of a randomized controlled trial
Source: BMC Geriatr. 2022 Sep 10;22:738. doi: 10.1186/s12877-022-03435-3 (PMC9463852; doi:10.1186/s12877-022-03435-3)
Supplement: Supplementary file 1 — Additional file 1: Table S1. Detailed exercise protocol for each group. [file 12877_2022_3435_MOESM1_ESM.docx]

**Electronic Supplementary Material Table 1.** Detailed exercise protocol for each group

|  | Trunk strengthening | Walking-balance |
| --- | --- | --- |
| Description of Exercises per Group | - **Trunk strengthening/motor control exercises:** (e.g., abdominal bracing, quadruped pose, curl-up, supine bridge, and side bridge) - **Walking-balance exercises**: Otago balance exercises [(e.g., calf/toe raises, toe/heel walking, backwards walking, heel toe walking backwards, figure 8 walking, sit to stand from chair, knee bends (squad), leg lift] and outdoor walking | - **Walking-balance exercises:** Otago balance exercises [(e.g., calf/toe raises, toe/heel walking, backwards walking, heel toe walking backwards, figure 8 walking, sit to stand from chair, knee bends (squad), leg lift] and outdoor walking |
| Traning volume | - 12-week exercise program with a total of 36 sessions - Each exercise session lasted 60 min - **Trunk strengthening/motor control exercises (30 min)**   - 5 exercises   - Initial dose (4 rep of 4 sec contraction time)   - Goal (8 rep of 8 sec contraction time)   - 30 sec of rest between reps and 2-3 min between exercises - **Walking-balance exercises (30 min)**   - Otago balance exercises (15 min)   - Continuous outdoor walking (15 min) | - 12-week exercise program with a total of 36 sessions - Each exercise session lasted 60 min - **Walking-balance exercises (60 min)**   - Otago balance exercises (15 min)   - Continuous outdoor walking (45 min) |
| Training frequency | - Three supervised training sessions per week (non-consecutive days) | - Three supervised training sessions per week (non-consecutive days) |
| Progression of Training Intensity for Various Exercises | - **Trunk strengthening/motor control exercises**   - from easy to challenging by changing the lever lengths (e.g., from bent knees side bridge to side bridge with one leg straight), range of motion (e.g., modified beginner curl-up on the wedge to beginner curl-up from the floor), movement velocity (e.g., static quadruped opposite arm/leg lifts to dynamic quadruped opposite arm/leg lifts) and the level of stability/instability (e.g., side bridge with one leg straight to side bridge with one leg extended and forearm on Airex Balance Pad). - **Walking-balance exercises**   - **Otago balance exercises** from easy to challenging by reducing base of support (using chair, wall, normal stance without support, semi tandem, tandem, one legged stance, shifting weight, changing from static to dynamic, and changing walk/gait direction, using unstable surfaces (e.g., Airex Balance Pad, Swiss ball )   - **Continuous outdoor walking** at approximately 60% of participants’ maximum heart rate using the age-based prediction formula [(220- age) - (resting heart rate)) × (60 %) + (resting heart rate)] | - **Walking-balance exercises**   - **Otago balance exercises** from easy to challenging by reducing base of support (using chair, wall, normal stance without support, semi tandem, tandem, one legged stance, shifting weight, changing from static to dynamic, and changing walk/gait dirction, using unstable surfaces (e.g., Airex Balance Pad, Swiss ball )   - **Continuous outdoor walking** at approximately 60% of participants’ maximum heart rate using the age-based prediction formula [(220- age) - (resting heart rate)) × (60 %) + (resting heart rate)] |
| Note. Adapted from “Trunk exercise training improves muscle size, strength, and function in older adults: A randomized controlled trial”, by Shahtahmassebi, B., Hebert, J. J., Hecimovich, M., & Fairchild, T. J (2019), *Scandinavian journal of medicine & science in sports*, 29(7), 980-991. <https://doi.org/10.1111/sms.13415> | | |
