# Supplementary material for: The effect of the inclusion of trunk-strengthening exercises to a multimodal exercise program on physical activity levels and psychological functioning in older adults: secondary data analysis of a randomized controlled trial
Source: BMC Geriatr. 2022 Sep 10;22:738. doi: 10.1186/s12877-022-03435-3 (PMC9463852; doi:10.1186/s12877-022-03435-3)
Supplement: Supplementary file 2 — Additional file 2: Table S2. Accumulated accelerometer valid wear time. [file 12877_2022_3435_MOESM2_ESM.docx]

| **Exercise groups** | **Accelerometer Valid Wear Time** | | |
| --- | --- | --- | --- |
|  | **N (%)** | **Valid days** | **Valid hours per day** |
| **Trunk strengthening** | | | |
| Baseline | 32 (100%) | 6.38 ± 0.41 | 19.1 ± 1.24 |
| 6 weeks | 27 (84.3%) | 6.28 ± 0.69 | 18.8 ± 2.08 |
| 12 weeks | 27 (84.3%) | 6.32 ± 0.61 | 18.9 ± 1.85 |
| 18 weeks | 27 (84.3%) | 6.34 ± 0.59 | 19.0 ± 1.79 |
| **Walking-balance** |  |  |  |
| Baseline | 32 (100%) | 6.39 ± 0.41 | 19.1 ± 1.24 |
| 6 weeks | 31 (96.8%) | 6.46 ± 0.34 | 19.3 ± 1.03 |
| 12 weeks | 30 (93.7%) | 6.41 ± 0.39 | 19.2 ± 1.17 |
| 18 weeks | 29 (90.6%) | 6.46 ± 0.43 | 19.3 ± 1.29 |
| ^*^The proportion of wear time (as percentage of total daily wear time) spent in sedentary behaviour, light physical activity; moderate physical activity; vigorous physical activity, and MPVA was calculated by dividing the sum of time for a given outcome (minutes per day) by total valid wear time (minutes per day).  Values are presented as Mean ± SD. Participants should accumulate at least 10 hours wear time per day on at least four days to be included in data analysis. N: number of participants, %: percentage | | | |

**Electronic Supplementary Material Table 2.** Accumulated accelerometer valid wear time* (numbers days and hours per day)
