# Supplementary material for: The effect of the inclusion of trunk-strengthening exercises to a multimodal exercise program on physical activity levels and psychological functioning in older adults: secondary data analysis of a randomized controlled trial
Source: BMC Geriatr. 2022 Sep 10;22:738. doi: 10.1186/s12877-022-03435-3 (PMC9463852; doi:10.1186/s12877-022-03435-3)
Supplement: Supplementary file 3 — Additional file 3: Table S3. The proportion of wear time spent in sedentary behavior and different intensity levels of physical activity. [file 12877_2022_3435_MOESM3_ESM.docx]

**Electronic Supplementary Material Table 3.** The proportion of wear time spent in sedentary behavior and different intensity levels of physical activity

| **Exercise groups** | **% of total daily wear time (Mean ± SD)** | | | | |
| --- | --- | --- | --- | --- | --- |
|  | **Sedentary time** | **Light physical activity** | **Moderate physical activity** | **Vigorous physical activity** | **MVPA** |
| **Trunk strengthening** | | | | | |
| Baseline (n=32) | 76.7 ± 5.66 | 21 ± 3.99 | 2.10 ± 2.96 | 0.03 ± 0.13 | 2.15 ± 3.10 |
| 6 weeks (n=27) | 76.2 ± 7.00 | 21.1 ± 4.47 | 2.53 ± 3.6 | 0.008 ± 0.01 | 2.46 ± 3.56 |
| 12 weeks (n=27) | 76.1 ± 6.81 | 20.9 ± 4.86 | 2.85 ± 2.92 | 0.01 ± 0.03 | 2.86 ± 2.93 |
| 18 weeks (n=27) | 76.0 ± 7.83 | 21.7 ± 6.5 | 2.22 ± 2.41 | 0.005 ± 0.01 | 2.23 ± 2.41 |
| **Walking-balance** | | | | | |
| Baseline (n= 32) | 76.8 ± 4.79 | 21.3 ± 4.21  4.79)  4.91)  4.36) | 1.69 ± 1.52 | 0.08 ± 0.28 | 1.77 ± 1.59 |
| 6 weeks (n= 31) | 75.2 ± 5.57 | 22 ± 4.79 | 2.59 ± 1.69 | 0.09 ± 0.19 | 2.68 ± 1.70 |
| 12 weeks (n=30) | 76.6 ± 5.36 | 21 ± 4.91  4.36) | 2.11 ± 1.37 | 0.10 ± 0.32 | 2.22 ± 1.14 |
| 18 weeks (n=29) | 76.1 ± 5.26 | 21.6 ± 4.36 | 2.15 ± 1.66 | 0.04 ± 0.11 | 2.20 ± 1.69 |
| Values are presented as percentage of total daily wear time (Mean ± SD). The proportion of wear time (as percentage of total daily wear time) spent in sedentary behaviour, light physical activity; moderate physical activity; vigorous physical activity, and MPVA was calculated by dividing the sum of time for a given outcome (minutes per day) by total valid wear time (minutes per day). MVPA: moderate-to-vigorous physical activity | | | | | |
