# Supplementary material for: The effect of the inclusion of trunk-strengthening exercises to a multimodal exercise program on physical activity levels and psychological functioning in older adults: secondary data analysis of a randomized controlled trial
Source: BMC Geriatr. 2022 Sep 10;22:738. doi: 10.1186/s12877-022-03435-3 (PMC9463852; doi:10.1186/s12877-022-03435-3)
Supplement: Supplementary file 4 — Additional file 4: Table S4. Changes in physical activity and sedentary behavior in response to the exercise program and detraining. [file 12877_2022_3435_MOESM4_ESM.docx]

**Electronic Supplementary Material Table 4.** Changes^a^ in physical activity and sedentary behaviour in response to the exercise program and detraining

| **Outcome measures, unit (Group** **X Time Interaction, *P-*values)** | **Trunk strengthening exercise group (n=32)** | **Walking-balance exercise group (n=32)** | **Mean between-group difference (95% CI):Trunk strengthening vs. Walking-balance exercise group** | ***P*-values** |
| --- | --- | --- | --- | --- |
| **Wear time, min/day (*P*= 0.58)** | | | | |
| Baseline | 1148.7 (74.8) | 1151.8 (74.9) | -3.016 (-45.6 to 39.6) | 0.88 |
| 6 weeks | 1129.5 (125.1) | 1162.4 (62.0) | -33.3 (-84.6 to 17.8) | 0.19 |
| 12 weeks | 1142.7 (111.1) | 1154.3 (70.6) | -11.7 (-56.6 to 33.2) | 0.60 |
| 18 weeks | 1137.5 (107.4) | 1162.1 (77.7) | -24.8 (-69.4 to 19.7) | 0.27 |
| **Mean difference (95% CI): Baseline vs. week 6** | -19.4 (-55.2 to 16.3) | 10.9 (-22.9 to 44.7) |  |  |
| **Mean difference (95% CI): Baseline vs. week 12** | -6.35 (-37.1 to 24.4) | 2.34 (-27.2 to 31.9) |  |  |
| **Mean difference (95% CI): Baseline vs. week 18** | -10.9 (-41.3 to 19.3) | 10.8 (-18.6 to 40.3) |  |  |
| **Mean difference (95% CI): Week 12 vs. week 18** | -4.64 (-36.8 to 27.6) | 8.48 (-22.1 to 39.1) |  |  |
| **Sedentary time, min/day (*P=* 0.54)** | | | | |
| Baseline | 888.3 (94.5) | 883.8 (67.1) | 4.52 (-43.1 to 52.1) | 0.84 |
| 6 weeks | 827.9 (175.6) | 874.9 (82.5) | -47.2 (-114.1 to 20.1) | 0.16 |
| 12 weeks | 848.1 (141.0) | 864.4 (142.0) | -16.2 (-84.6 to 52.1) | 0.63 |
| 18 weeks | 862.4 (131.3) | 879.8 (75.1) | -17.3 (-71.5 to 36.8) | 0.52 |
| **Mean difference (95% CI): Baseline vs. week 6** | -60.3 (-111.2 to -9.4) | -8.83 (-58.3 to 40.7) |  |  |
| **Mean difference (95% CI): Baseline vs. week 12** | -40.1 (-92.3 to 12.1) | -19.4 (-69.4 to 30.6) |  |  |
| **Mean difference (95% CI): Baseline vs. week 18** | -25.8 (-68.0 to 16.3) | -4.00 (-45.1 to 37.1) |  |  |
| **Mean difference (95% CI): Week 12 vs. week 18** | 14.321 (-41.6 to 70.2) | 15.4 (-37.8 to 68.6) |  |  |
| **Overall physical activity level (average** **counts per minute counts); CPM (*P*= 0.057)** | | | | |
| Baseline | 180.9 (129.9) | 174.1 (81.1) | 6.75 (-55.5 to 69.0) | 0.82 |
| 6 weeks | 192.9 (186.1) | 210.3 (87.7) | -17.3 (-80.3 to 45.5) | 0.58 |
| 12 weeks | 209.7 (167.6) | 188.9 (74.0) | 20.7 (-41.0 to 82.5) | 0.50 |
| 18 weeks | 168.6 (156.8) | 180.0 (101.6) | -11.4 (-77.1 to 54.2) | 0.73 |
| **Mean difference (95% CI): Baseline vs. week 6** | 12.1 (-9.74 to 33.8) | 36.1 (14.8 to 57.4) |  |  |
| **Mean difference (95% CI): Baseline vs. week 12** | 28.8 (8.95 to 48.6) | 14.7 (-4.40 to 33.9) |  |  |
| **Mean difference (95% CI): Baseline vs. week 18** | -12.2 (-37.6 to 13.0) | 5.86 (-19.4 to 31.1) |  |  |
| **Mean difference (95% CI): Week 12 vs. week 18** | -41.1 (-66.4 to -15.7) | -8.90 (-33.6 to 15.8) |  |  |
|  |  |  |  |  |
| **Table 4 continued** | | | | |
| **Outcome measures, unit (Group** **X Time Interaction, *P-*values)** | **Trunk strengthening exercise group (n=32)** | **Walking-balance exercise group (n=32)** | **Mean between-group difference (95% CI):Trunk strengthening vs. Walking-balance exercise group** | ***P*-values** |
| **Total number of steps per min/day, number (*P*= 0.009)** | | | | |
| Baseline | 4.96 (1.67) | 5.06 (1.86) | -0.10 (-1.04 to 0.84) | 0.82 |
| 6 weeks | 5.19 (2.23) | 6.22 (1.96) | -1.02 (-1.99 to -0.06) | 0.03 |
| 12 weeks | 5.78 (2.28) | 5.55 (1.79) | 0.22 (-0.75 to 1.20) | 0.65 |
| 18 weeks | 5.07 (1.72) | 5.51 (1.93) | -0.43 (-1.42 to 0.55) | 0.38 |
| **Mean difference (95% CI): Baseline vs. week 6** | 0.22 (-0.27 to 0.73) | 1.15 (0.65 to 1.64) |  |  |
| **Mean difference (95% CI): Baseline vs. week 12** | 0.81 (0.29 to 1.33) | 0.49 (-0.01 to 0.99) |  |  |
| **Mean difference (95% CI): Baseline vs. week 18** | 0.11 (-0.40 to 0.63) | 0.44 (-0.06 to 0.95) |  |  |
| **Mean difference (95% CI): Week 12 vs. week 18** | -0.70 (-1.25 to -0.15) | -0.04 (-0.58 to 0.48) |  |  |
| **Light physical activity, min/day (*P*= 0.43)** | | | | |
| Baseline | 246.4 (43.5) | 256.3 (48.9) | -9.95 ( -39.4 to 19.5) | 0.501 |
| 6 weeks | 229.5 (67.6) | 256.6 (54.0) | -27.9 (-56.7 to 2.56) | 0.07 |
| 12 weeks | 234.1 (63.9) | 238.4 (64.6) | -6.415 (-37.1 to 24.2) | 0.67 |
| 18 weeks | 240 (63.8) | 253.0 (56.2) | -13.4 ( -42.0 to 15.2) | 0.35 |
| **Mean difference (95% CI): Baseline vs. week 6** | -16.827 (-35.3 to1.66) | 0.30 (-17.7 to 18.4) |  |  |
| **Mean difference (95% CI): Baseline vs. week 12** | -14.301 (-33.7 to 5.1) | -17.8 (-36.6 to 0.95) |  |  |
| **Mean difference (95% CI): Baseline vs. week 18** | -6.420 (-24.1 to 11.2) | -2.96 (-20.2 to 14.3) |  |  |
| **Mean difference (95% CI): Week 12 vs. week 18** | 7.88 (-11.2 to 27.0) | 14.8 (-3.24 to 33.00) |  |  |
| **Moderate physical activity, min/day (*P=* 0.01)** | | | | |
| Baseline | 23.9 (30.5) | 19.5 (17.6) | 4.39 ( -8.83 to 17.6) | 0.49 |
| 6 weeks | 26.2 (40.8) | 30.1 (20.4) | -3.91 (-17.2to 9.39) | 0.56 |
| 12 weeks | 30.2 (33.5) | 23.7 (16.7) | 6.46 (-6.86 to 19.8) | 0.33 |
| 18 weeks | 25.7 (27.9) | 25.4 (20.3) | -2.57 (-15.9 to 10.8) | 0.68 |
| **Mean difference (95% CI): Baseline vs. week 6** | 2.30 (-2.57 to 7.15) | 10.6 (5.74 to 15.4) |  |  |
| **Mean difference (95% CI): Baseline vs. week 12** | 6.29 (1.58 to 11.00) | 4.19 (-0.67 to 9.06) |  |  |
| **Mean difference (95% CI): Baseline vs. week 18** | -0.91 (-5.65 to 3.81) | 5.90 (0.92 to 10.8) |  |  |
| **Mean difference (95% CI): Week 12 vs. week 18** | -7.21 (-12.8 to -1.59) | 1.71 (-3.26 to 6.69) |  |  |
|  |  |  |  |  |
| **Table 4 continued** |  |  |  |  |
| **Outcome measures, unit (Group** **X Time Interaction, *P-*values)** | **Trunk strengthening exercise group (n=32)** | **Walking-balance exercise group (n=32)** | **Mean between-group difference (95% CI):Trunk strengthening vs. Walking-balance exercise group** | ***P*-values** |
| **Vigorous physical activity, min/day (*P*= 0.64)** | | | | |
| Baseline | 0.36 (1.35) | 0.94 (3.25) | -0.58 (-1.79 to 0.63) | 0.34 |
| 6 weeks | 0.09 (0.21) | 1.00 (2.20) | -0.90 (-1.73 to -0.08) | 0.03 |
| 12 weeks | 0.15 (0.38) | 1.21 (3.64) | -1.05 (-2.35 to 0.25) | 0.11 |
| 18 weeks | 0.07 (0.20) | 0.48 (1.25) | -0.40 (-0.89 to 0.073) | 0.09 |
| **Mean difference (95% CI): Baseline vs. week 6** | -0.27 -1.30 to 0.76) | 0.05 (-0.96 to 1.07) |  |  |
| **Mean difference (95% CI): Baseline vs. week 12** | -0.21 (-1.45 to 1.03) | 0.26 (-0.98 to 1.50) |  |  |
| **Mean difference (95% CI): Baseline vs. week 18** | -0.29 (-1.21 to 0.62) | -0.46 (-1.38 to 0.45) |  |  |
| **Mean difference (95% CI): Week 12 vs. week 18** | -0.082 (-1.06 to 0.89) | -0.727 (-1.70 to 0.25) |  |  |
| **Moderate-to-vigorous physical activity (MVPA), min/day (*P*= 0.004)** | | | | |
| Baseline | 26.7 (34.8) | 20.5 (18.4) | 6.12 (-7.51 to 19.7) | 0.37 |
| 6 weeks | 26.2 (40.9) | 31.2 (20.5) | -4.97 (-18.8 to 8.78) | 0.47 |
| 12 weeks | 31.4 (33.5) | 25.3 (17.2) | 6.07 (-7.05 to 19.2) | 0.35 |
| 18 weeks | 22.1 (27.5) | 25.9 (20.7) | -3.51 (-17.4 to 10.4) | 0.61 |
| **Mean difference (95% CI): Baseline vs. week 6** | -0.45 (-6.38 to 5.47) | 10.6 (4.85 to 16.4) |  |  |
| **Mean difference (95% CI): Baseline vs. week 12** | 4.76 (-0.13 to 9.66) | 4.81 (0.06 to 9.56) |  |  |
| **Mean difference (95% CI): Baseline vs. week 18** | -4.55 (-10.4 to 1.35) | 5.07 (-0.69 to 10.8) |  |  |
| **Mean difference (95% CI): Week 12 vs. week 18** | -9.32 (-14.5 to -4.06) | 0.26 (-4.73 to 5.26) |  |  |
| ^a^All differences are adjusted for the baseline value of the outcome variables. Values are presented as mean (SD) or as mean difference (95% CI).  The physical activity and sedentary behavior assessments comprised measures obtained over seven consecutive days of accelerometer wear (ActiGraph GT3X). | | | | |
