# Supplementary material for: The effect of the inclusion of trunk-strengthening exercises to a multimodal exercise program on physical activity levels and psychological functioning in older adults: secondary data analysis of a randomized controlled trial
Source: BMC Geriatr. 2022 Sep 10;22:738. doi: 10.1186/s12877-022-03435-3 (PMC9463852; doi:10.1186/s12877-022-03435-3)
Supplement: Supplementary file 5 — Additional file 5: Table S5. Changes in psychological outcomes in response to the exercise program and detraining. [file 12877_2022_3435_MOESM5_ESM.docx]

**Electronic Supplementary Material Table 5.** Changes^a^ in psychological outcomes in response to the exercise program and detraining

| **Outcome measures (Group by Time Interaction, *P*-value)** | **Trunk strengthening exercise group (n=32)** | **Walking-balance exercise group (n=32)** | **Mean between-group difference (95% CI):Trunk strengthening vs. Walking-balance exercise group** | ***P*-values** |
| --- | --- | --- | --- | --- |
| **Falls Self-Efficacy Scale (FES-I) (*P*= 0.16)** | | | | |
| Baseline | 18.88 (3.47) | 19.09 (3.55) | -0.21 (-1.94 to 1.51) | 0.80 |
| 6 weeks | 18.34 (3.23) | 19.03 (2.87) | -0.68 (-2.34 to 0.97) | 0.41 |
| 12 weeks | 18.83 (3.91) | 18.62 (2.99) | 0.20 (-1.41 to 1.83) ) | 0.80 |
| 18 weeks | 17.97 (1.44) | 18.99 (3.70) | -1.02 (-2.70 to 0.65) | 0.22 |
| **Mean difference (95% CI): Baseline vs. week 6** | -0.53 (-1.47 to 0.41) | -0.06 (-0.98 to 0.85) |  |  |
| **Mean difference (95% CI): Baseline vs. week 12** | -0.04 (-0.94 to 0.86) | -0.46 (-1.34 to 0.41) ) |  |  |
| **Mean difference (95% CI): Baseline vs. week 18** | -0.90 (-1.86 to 0.05) | -0.09 (-1.02 to 0.82) |  |  |
| **Mean difference (95% CI): Week 12 vs. week 18** | -0.86 (-1.70 to -0.03) | 0.36 (-0.42 to 1.16) |  |  |
| **Geriatric Anxiety Inventory (GAI) (*P*= 0.80)** | | | | |
| Baseline | 1.06 (2.29) | 1.37 (2.19) | -0.31 (-1.42 to 0.79) | 0.57 |
| 6 weeks | 1.03 (2.65) | 1.30 (1.85) | -0.25 (-1.30 to 0.79) | 0.63 |
| 12 weeks | 0.75 (1.72) | 1.07 (1.53) | -0.31 (-1.15 to 0.51) | 0.45 |
| 18 weeks | 0.93 (1.94) | 0.97 (1.48) | -0.03 (-1.02 to 0.95) | 0.94 |
| **Mean difference (95% CI): Baseline vs. week 6** | -0.01 (-0.74 to 0.72) | -0.07 (-0.79 to 0.64) |  |  |
| **Mean difference (95% CI): Baseline vs. week 12** | -0.30 (-0.88 to 0.26) | -0.30 (-0.86 to 0.26) |  |  |
| **Mean difference (95% CI): Baseline vs. week 18** | -0.12 (-0.81 to 0.56) | -0.40 (-1.07 to 0.26) |  |  |
| **Mean difference (95% CI): Week 12 vs. week 18** | 0.18 (-0.23 to 0.60) | -0.10 (-0.49 to 0.29) |  |  |
| **Geriatric Depression Scale (GDS) (*P*= 0.87)** | | | | |
| Baseline | 0.40 (0.94) | 0.53 (0.62) | -0.12 (-0.52 to 0.27) | 0.53 |
| 6 weeks | 0.39 (0.94) | 0.45 (0.67) | -0.06 (-0.42 to 0.30) | 0.73 |
| 12 weeks | 0.25 (0.65) | 0.26 (0.44) | -0.006 (-0.29 to 0.28) | 0.96 |
| 18 weeks | 0.32 (0.73) | 0.36 (0.80) | -0.04 (-0.48 to 0.40) | 0.85 |
| **Mean difference (95% CI): Baseline vs. week 6** | -0.01 (-0.28 to 0.26) | -0.07 (-0.34 to 0.19) |  |  |
| **Mean difference (95% CI): Baseline vs. week 12** | -0.15 (-0.37 to 0.07) | -0.26 (-0.49 to -0.04) |  |  |
| **Mean difference (95% CI): Baseline vs. week 18** | -0.08 (-0.41 to 0.24) | -0.16 (-0.48 to 0.15) |  |  |
| **Mean difference (95% CI): Week 12 vs. week 18** | 0.06 (-0.20 to 0.33) | 0.10 (-0.15 to 0.35) |  |  |
| ^a^All differences are adjusted for the baseline value of the outcome variables. Values are presented as mean (SD) or as mean difference (95% CI). | | | | |
